# Association between Male Partner Involvement and the Uptake of Prevention of Mother-to-Child Transmission of HIV (PMTCT) Interventions in Mwanza District, Malawi: A Retrospective Cohort Study

**DOI:** 10.1371/journal.pone.0066517

**Published:** 2013-06-12

**Authors:** Fatch W. Kalembo, Maggie Zgambo, Atupele N. Mulaga, Du Yukai, Niman I. Ahmed

**Affiliations:** 1 Department of Child, Adolescence & Woman Health Care, School of Public Health, Tongji Medical College, Huazhong University of Science & Technology, Wuhan, China; 2 Department of Nursing, Xiangya Medical College, Central South University, Changsha, China; 3 Department of Mathematics and Statistics, The Polytechnic, University of Malawi, Blantyre, Malawi; Tulane University, United States of America

## Abstract

**Objective:**

The main objective of this study was to examine the association between male partner involvement and the uptake of prevention of mother-to-child transmission of HIV (PMTCT) interventions.

**Methods:**

A retrospective cohort study was used to collect data on women, their male partners and their children who were enrolled in a PMTCT program from January 2004 to December 2006 at Mwanza District Hospital. HIV infected women and their children were followed-up over the 18 months postnatal period. Data were analyzed using descriptive statistics, chi-square test and logistic regression.

**Results:**

A total of 476 HIV positive women were enrolled in a PMTCT program and were followed-up in the study. Of those followed-up in the study, 65 (13.7%) had a male partner involvement while 411 (86.3%) had no male partner involvement. Male partner involvement was significantly associated with condom use (Adjusted odds ratio [AOR] = 5.6, 95% confidence interval [CI]: 2.3–13.5, *P*<0.001), hospital delivery (AOR = 25.9, 95%CI: 10.6–63.6, *P*<0.001), and completion of follow-up in the program (AOR = 16.8, 95% CI: 8.5–33.4, *P*<0.001).

**Conclusion:**

Male partner involvement increases the uptake of some PMTCT interventions by HIV positive women. Multi-strategic, culturally tailored public health care models are needed to increase the rate of male partner involvement in the program.

## Introduction

Human immunodeficiency virus (HIV) remains a major challenge globally despite decades of advocacy and investment in programs to control the spread of the virus. UNAIDS estimated that in 2009, 33.3 million people globally were living with HIV. UNAIDS further estimated that about 2.5 million children globally were living with HIV and 1.8 million of these were from Sub-Saharan Africa [1]. In Malawi 11% of the estimated population of 15 million were living with HIV in 2009. This included 57,000 pregnant women and 120,000 children under the age of 14 [2].

Mother-to-child transmission (MTCT) is the most common route of HIV infection for HIV positive children under five years. It is believed that with effective PMTCT interventions, MTCT can be reduced to levels below 5% [3]. A PMTCT program consists of a range of interventions, including improved antenatal services, opt-out HIV counseling and testing for pregnant women, antiretroviral drug prophylaxis for HIV positive pregnant women and newborns, referral to support groups, counseling on options for safer infant feeding practices and continued follow-up and treatment for HIV positive mothers and their children for the first 18 months of the child’s life [3]–[4].

Men are the decision makers in many African countries where PMTCT is offered [5]. One major factor that prevents some women from accepting HIV testing is the need to seek their partners’ consent [6]. With a male partner involvement in PMTCT, a couple has a chance to make informed decisions together on living positively with HIV, to share responsibility for preventing HIV transmission to the unborn child and to discuss safer sex practices, as well as to make informed decisions to access care and treatment [7]. In addition, men can also play a crucial role in supporting HIV positive pregnant women, by assisting them to get to clinics or hospitals where chances of safe delivery are higher, and to choose a safe infant feeding method [8].

In 2003, the Government of Malawi introduced male partner involvement in PMTCT program. It was observed that before the introduction of the program, many pregnant women were shunning HIV testing because they did not have the consent of their husbands. Those who had the courage to go for the test and tested HIV positive were afraid to disclose their serostatus to their husbands. They thought their husbands would accuse them of infidelity, an issue which could lead to divorce. These factors resulted in low uptake of PMTCT services by HIV positive women. Male partner involvement in PMTCT was then introduced with an aim of combating these problems and consequently increasing uptake and outcomes of PMTCT interventions [4]. Mwanza was one of the pioneer districts to initiate male partner involvement in PMTCT program. The aim of this study was to examine the association between male partner involvement and uptake of PMTCT interventions by HIV pregnant women at Mwanza District Hospital.

## Materials and Methods

### Study Setting

Mwanza district hospital is situated at the center of Mwanza district, which is located 198 km South-East of Lilongwe, the capital city of Malawi. The hospital has a catchment population of 48799 with 11224 women within the reproductive age group of 15 to 49. It provides services to an estimated number of 2840 pregnant women every year. The hospital is situated near the border between Malawi and Mozambique, so it also receives patients from Mozambique.

### Description of the PMTCT Program at Mwanza District Hospital

The point of entry in PMTCT service at Mwanza District Hospital during the study period (2004–2006) was through the antenatal clinic. Pregnant women were encouraged to come with their male partners to the antenatal clinic. Women without male partners were counseled individually in private rooms by midwives. Those who came with their male partners were counseled together as a couple. After counseling, HIV testing was done on those who consented. The Determine HIV-1/2 (Abbott Laboratories, Abbott Park, Illinois, USA) rapid test kit was being used to determine women’s HIV serostatus. A confirmatory Capillus HIV-1/HIV-2 (Cambridge Biotechnology, Galway, Ireland) rapid test was performed for all positive Determine results.

Post-test counseling was done within one hour after HIV testing. A code indicating the woman’s HIV status was put at the end of her antenatal record book. HIV-infected women who had completed 26 weeks of gestation were given a 200 mg Nevirapine (NVP) tablet, with instructions to take it at the onset of labor. Women who had not completed 26 weeks of gestation were given the tablet when they reached gestational age or upon presentation in labor. Infants born to the HIV mothers were given NVP syrup within 72 hours of delivery. After delivery, women were encouraged to return to the hospital after seven days and again at six weeks post-delivery, for continued care and support. Thereafter, the mother-infant pairs were asked to come for follow-up visits every 3 months until infant HIV diagnosis was made at 18 months of age.

### Study Design

A retrospective cohort study was conducted using data obtained from a routine PMTCT program implemented according to national guidelines.

### Study Population

The study population included HIV positive women with or without male partner involvement, and their infants who were enrolled in PMTCT program, within the period of 1^st^ January 2004 to 31^st^ December 2006.

### Data Collection and Flow of the Study Participants

We used a retrospective cohort study to examine the association between male partner involvement and the uptake of PMTCT interventions by HIV positive women. The PMTCT records from 1^st^ January 2004 to 31^st^ December 2006, of all pregnant women attending their first antenatal care visit at Mwanza District Hospital, were examined. A data extraction tool was designed to collect study participants’ variables from PMTCT registers. General antenatal data were obtained from the PMTCT antenatal register. The register contained information on all pregnant women and their partners who attended antenatal care from 2004 to 2006. From the antenatal register, we extracted the number of pregnant women who attended antenatal clinic, number of pregnant women who tested for HIV, the number of pregnant women who tested HIV positive, the number of pregnant women who came with their male partners, the number of male partners who tested for HIV and the number of male partners who tested HIV positive. All pregnant women who tested HIV positive were enrolled in the PMTCT program and subsequently recruited in the study.

The women who were recruited in the study were divided into two groups; the first group, with male partner involvement and the second one, without. Male partner involvement was described as a male partner accompanying his pregnant spouse to antenatal clinic, receiving counseling for HIV either together as a couple or individually, disclosing HIV results to each other and supporting his pregnant partner in adhering to PMTCT protocols. A man was considered to be supporting his partner to adhere to PMTCT protocols if he provided financial assistance for his pregnant spouse’s hospital visits or if he participated with his partner in decision making regarding the use of condoms and the choice of infant feeding method. The baseline criterion for consideration of male partner involvement in the study was male partner attendance at antenatal clinic with his partner. In addition to the baseline criterion, male partner involvement was also considered if a couple met any other component of the definition of male partner involvement in PMTCT program. The study participants were followed from antenatal period through labor and delivery to 18 months postnatally.

For the first antenatal visit, we extracted the following information from the PMTCT antenatal register for each study participant: age, educational level, marital status, occupational status, condom use and male partner involvement status. During the subsequent antenatal visit we obtained information on the consistency of condom use for each study participant. During labor and delivery, we extracted the following information: the study participants who delivered at the hospital, their mode of delivery, intake of ARV prophylaxis (single dose Nevirapine) for the mother and intake of ARV prophylaxis for infants within 72 hours of delivery. We used each study participant’s antenatal name and registration number to obtained labor and delivery information from labor and delivery PMTCT registers. During the postnatal period, study participants were required to come for follow-up visits; the first follow-up visit at 7 days after delivery, then at 6 weeks after delivery. Thereafter mother and infant pairs were seen every 3 months until the children were 18 months old. At each scheduled follow-up visit, we obtained the following information from the PMTCT registers: infant’s feeding practice, morbidity and mortality. Data for postnatal follow-up were extracted from postnatal PMTCT registers. At 18 months of age, children were tested for HIV using rapid HIV antibody test kits.

### Study Inclusion Criteria

In order for the subjects to be included in the study, they had to meet the following conditions: (i) were residents of Mwanza District; (ii) were enrolled in a PMTCT program during the antenatal period within the period of 1^st^ January, 2004 to 31^st^ December 2006; (iii) attended antenatal care at Mwanza district hospital; (iv) were initial antenatal care attendees and (v) were HIV positive.

### Study End Points

The study end point for condom use, mode of delivery and maternal ARV prophylaxis was by delivery while the end points for infant ARV prophylaxis, infant feeding option and mother-infant follow-up were by 72 hours post-delivery, six months postnatal period and 18 months postnatal period, respectively. At the 18 months postnatal period, children’s health outcomes and HIV status were determined. The outcomes were either HIV infected, uninfected or dead.

### Statistical Analysis

The Statistical Package for Social Sciences (SPSS version 18.0 for Windows; SPSS Inc., Chicago, IL, USA) was used for data analysis. Descriptive statistics were used to analyze the percentages of variables of the study participants. Pearson’s chi-square test was used to assess the difference between categorical variables. To better understand the association between male partner involvement and uptake of PMTCT interventions, multivariate logistic regression models were constructed and odds ratios (OR) were calculated to determine the direction and strength of the association. Variables found to be significant (*P*<0.05) in the univariate analyses were entered in the multiple logistic regression models with all significant potential confounders. The backward stepwise procedure was used to determine a final model. The final model retained independent variables that were statistically significant at the *P*<0.05 level. Study participants’ educational level and occupational status were retained as significant confounders in the final model. *P*-value <0.05 was considered significant for all analyses in the study.

### Ethics Statement

This study was approved by Huazhong University of Science and Technology Research Committee and Malawi National Health Science Research Committee. Further consent to carry out the study was obtained from the District Health Officer of Mwanza District Hospital. Our study used routinely collected data from hospital records and did not involve contact with study subjects. As such it was considered exempt from a full human subjects review and from obtaining informed consent. Both institutional review boards (Huazhong University of Science and Technology Research Committee and Malawi National Health Science Research Committee) waived the need for written informed consent from the participants. In order to ensure privacy of the study participants we used identification numbers rather than the names of the study subjects in processing the research data.

## Results

### Flow of Study Participants

**Figure 1:**
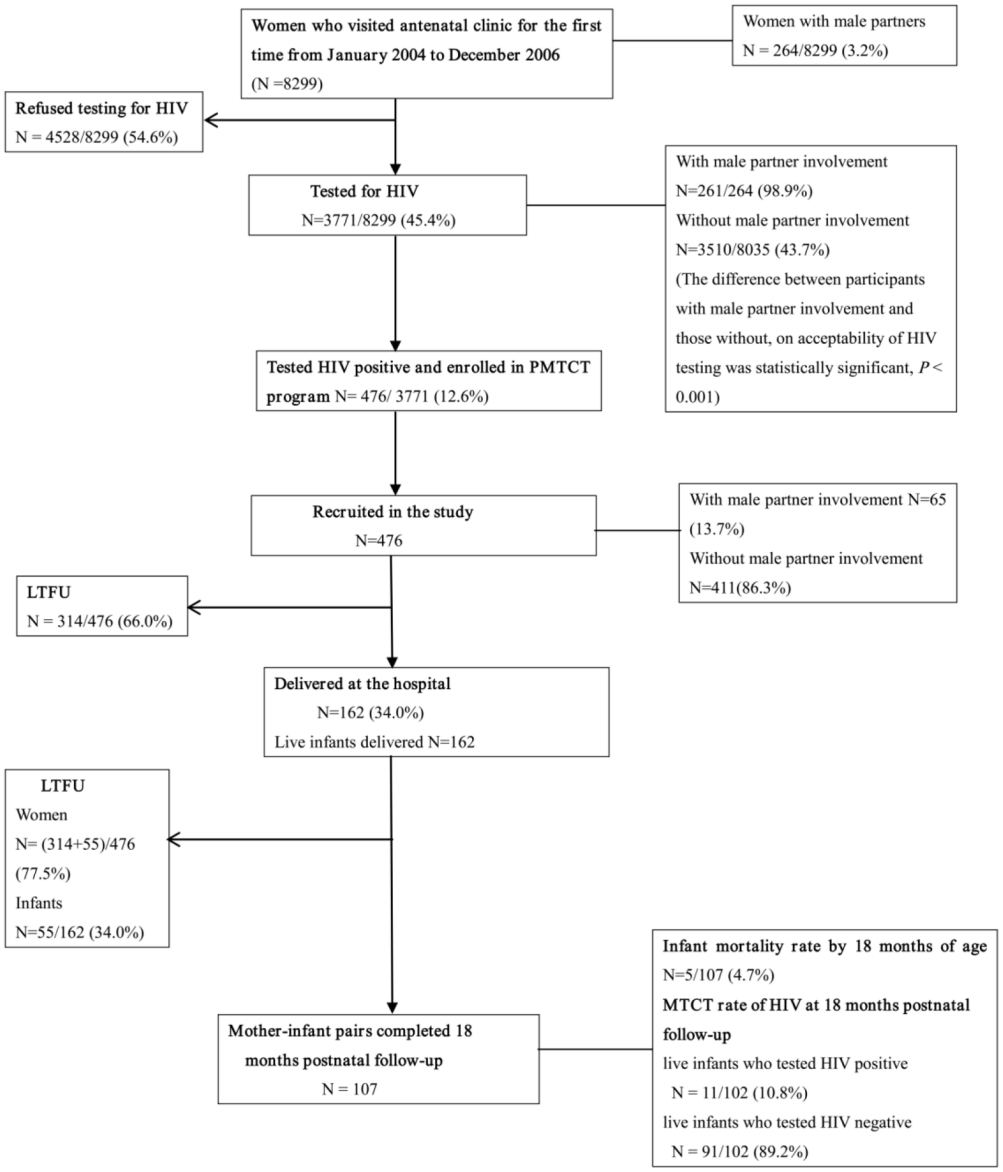
Flow of study participants. The flow of study participants in PMTCT program from January 2004 to December 2006 at Mwanza District Hospital. The study participants were followed-up from antenatal period, through labor and delivery, to 18 months postnatal period.

A total of 8299 women visited an antenatal clinic for the first time within the period of January 2004 and December 2006. Of these 264 (3.2%) were accompanied by their male partners. Less than half of the women 3771(45.4%) agreed to be tested for HIV and 476 (12.6%) of them tested HIV positive and were subsequently enrolled in the PMTCT program. Women who were accompanied by male partners had a higher acceptability of HIV testing than those without, 98.9% compared to 43.7% respectively, *P*<0.001. Four hundred and seventy-six HIV positive women, who were enrolled in the PMTCT program, were included in our study and of these, 65 (13.7%) had male partner involvement. A total of 314 (66%) women were lost to follow-up before delivery. The remaining 162 women delivered at the hospital to 162 live infants. During postnatal follow-up, 55 mother-infant pairs were lost to follow-up. A total of 107 mother-infant pairs completed the 18 months postnatal follow-up in the program. Among the infants who completed the 18 months follow-up in the program, 5 died, 91 were alive and tested HIV negative while 11 were alive and tested HIV positive (Figure 1).

### Association between Socio-demographic Characteristics and Male Partner Involvement

Univariate analysis revealed that women who attained primary, secondary and tertially education were likely to have male partner involvement (Odds ratio [OR] = 3.8, 95% confidence interval [CI]: 2.0–7.2, *P*<0.001), (OR = 4.2, 95% CI: 2.8–13.5, *P*<0.001) and (OR = 2.7, 95% CI: 1.4–7.9, *P*<0.001), respectively. Furthermore, women who were working or involved in business were significantly associated with male partner involvement (OR = 4.3, 95% CI: 2.3–7.8, *P*<0.001) (Table 1).

**Table 1:** Univariate associations between socio-demographic factors and male partner involvement in PMTCT.

Variables	Male involvement	No male involvement	OR	95% CI	*P*-value
	N (%)	N (%)			
	N = 65	N = 411			
**Age**					
14–24	44 (67.8)	217 (52.8)	1.0		
25–35	19 (29.2)	168 (40.9)	0.6	0.3–1.0	0.058
36–46	2 **(**3.0)	26 (6.3)	0.4	0.1–1.6	0.184
**Education**					
No education	15 (23.1)	226 (55.0)	1.0		
Primary	32 (49.2)	128 (31.1)	3.8	2.0–7.2	<0.001*
Secondary	12 (18.5)	39 (9.5)	4.2	2.8–13.5	<0.001*
Tertially	6 **(**9.2)	18 (4.4)	2.7	1.4–7.9	<0.001*
**Marital status**					
Unmarried	65(100.0)	399 (97.1)	1.0		
Married	0 (0.0)	12 (2.9)	1.0	0.9–1.6	0.163
**Occupation**					
No work/business	22(33.8 )	44 (10.7)	1.0		
With work/business	43(66.2)	367 (89.3)	4.3	2.3–7.8	<0.001*

* *P*-value significant at 0.05.

1.0: Reference category.

OR: Odds ratio.

95%CI: 95 percent confidence interval.

### Association between Male Partner Involvement and Uptake and Outcomes of PMTCT Interventions

In univariate analysis, male partner involvement was significantly associated with condom use (OR = 8.3, 95%CI: 3.5–19.6, *P*<0.001), artificial infant feeding (OR = 3.1, 95%CI: 1.6–6.2, *P*<0.01), hospital delivery (OR = 29.4, CI: 12.3–70.1, *P*<0.001) and completion of follow-up in the program (OR = 19.0, CI: 10.1–35.7, *P*<0.001) (Table 2).

**Table 2:** Univariate associations between male partner involvement and uptake and outcomes of PMTCT interventions.

Variables	Male involvement	No male involvement	OR	95%CI	*P*-Value
	N (%)	N (%)			
**Uptake of PMTCT**	N = 65	N = 411			
**Condom use** a					
Did not use condoms	5 (7.7)	186 (45.3)	1.0		
Used condoms	60(92.3)	225 (54.7)	8.3	3.5–19.6	<0.001*
**Place of delivery** a					
Not at the hospital	5(7.7)	309(72.2)	1.0		
Hospital	60(92.3)	102(27.8)	29.4	12.3–70.1	<0.001*
**Maternal ARV prophylaxis** a					
Did not take Nevirapine	10 (16.7)	26 (25.5)	1.0		
Took Nevirapine (NVP)	50 (83.3 )	76 (74.5)	1.6	0.7–3.7	0.240
**Mode of delivery** a					
SVD	50 (83.3)	91(89.2)	1.0		
CS	10 (16.7)	10 (9.8)	1.9	0.7–5.0	0.217
**Infant ARV prophylaxis** a					
Did not take NVP	7 (11.7)	14 (13.7)	1.0		
Took NVP	51(85.0)	86 (84.3)	1.4	0.5–3.8	0.556
**Infant feeding option** a					
EBF	38 (63.3)	75 (73.5)	1.0		
AF	22 (36.7)	27 (26.5)	3.1	1.6–6.2	0.01*
**Follow-up** a					
Loss to follow- up	17 (26.1)	352(85.6)	1.0		
Completed follow-up	47 (72.3)	55(13.4)	19.0	10.1–35.7	<0.001*
**Outcomes of PMTCT Child health outcomes** b	N = 60	N = 102			
Dead	1 (1.7)	4 (3.9)	1.0		
Alive	47 (78.3)	55 (53.9)	2.3	0.3–21.5	0.451
**Infant HIV status** b					
Positive	4 (8.5)	7(12.5)	1.0		
Negative	43 (91.5)	48 (87.3)	1.8	0.4–7.5	0.438

a Uptake of PMTCT intervention,

b outcomes of PMTCT interventions, OR: Odds ratio, 95% CI: 95 percent confidence interval,

* *P*<0.05, 1.0: Reference category.

In the final model of multivariate analysis, male partner involvement was significantly associated with condom use (Adjusted odds ratio [AOR] = 5.6, 95% confidence interval [CI]: 2.3–13.5, *P*<0.001), hospital delivery (AOR = 25.9, 95%CI: 10.6–63.6, *P*<0.001), and completion of follow-up in the program (AOR = 16.8, 95% CI: 8.5–33.4, *P*<0.001) (Table 3).

**Table 3:** Multivariate associations between male partner involvement and uptake of PMTCT interventions.

Variable	Male involvement N (%)	No male involvement N (%)	AOR	95%CI	*P*-Value
	N = 65	N = 411			
**Condom use**					
Did not use condoms	5 (7.7)	186 (45.3)	1.0		
Used condoms	60(92.3)	225 (54.7)	5.6	2.3–13.5	<0.001*
**Place of delivery**					
Not at the hospital	5(7.7)	309(72.2)	1.0		
Hospital	60(92.3)	102(27.8)	25.9	10.6–63.6	<0.001*
**Infant feeding option**					
EBF	38 (63.3)	75 (73.5)	NS		
AF	22 (36.7)	27 (26.5)			
**Follow-up**					
Loss to follow- up	17 (26.1)	352(85.6)	1.0		
Completed follow-up	47 (72.3)	55(13.4)	16.8	8.5–33.4	<0.001*

Adjusted for women’s educational level and occupational status.

* *P*<0.05.

1.0: Reference category.

AOR: Adjusted odds ratio.

95%CI: 95 percent confidence interval.

NS: Variable included in multivariate analysis but not significant in the final model.

## Discussion

In our study, 45.4% of the women who came for antenatal care consented to be tested for HIV and 12.6% of them tested HIV positive. The number of women who consented to HIV testing in this study was low. This is a lost opportunity for those who refused to be tested to know their HIV status and to protect their unborn babies from HIV infection. The low HIV testing rate may be attributed to many factors. Studies have suggested low male partner involvement in PMTCT, stigma and discrimination, and fear of testing positive for HIV as reasons for refusing to test for HIV [9]–[10].

The rate of male partner involvement in PMTCT in our study was 3.2% which is far below the rates in studies conducted in other parts of Sub-Saharan Africa where the PMTCT program is being implemented. For example, male partner attendance rates in northern Tanzania, Kenya, Uganda and Ivory Coast were at 12.5%, 16%, 65.4% and 25%, respectively [11]–[14]. The low rate of male partner involvement in this study implies that, if the women tested HIV positive, it would be difficult for them to get full support and encouragement from their male partners. Culturally, in Malawi, women are not permitted to communicate HIV status and initiate condom use with their spouses because this is associated with women having extra marital affairs [15]. The findings of this study highlight the need for government and health workers to create a conducive environment in antenatal clinics for male partners’ participation. This strategy was found to be effective in Burkina Faso and Malawi [16]–[17].

Our study found that there were significant differences in the uptake of PMTCT interventions. Subjects with male partner involvement were more likely to use condoms, deliver at the hospital and complete follow-up in the program compared to those without male partner involvement. The findings of our study are similar to the findings of several studies in sub-Saharan Africa where male partner involvement was associated with a higher likelihood of women implementing PMTCT interventions [18]–[21]. The results of our study emphasize the importance of male partners in promoting the uptake of PMTCT interventions by women. In contrast to the findings of our study, a study in Cambodia found that uptake of PMTCT interventions was not influenced by a male partner involvement but rather by maternal basic knowledge of HIV [22].

The findings of this study have shown that women with a male partner involvement were more likely to have infants who tested HIV negative than those without male partner involvement. However, the association between male partner involvement and infant HIV status was statistically insignificant (OR = 1.8; 95% CI: 0.4–7.5, *P* = 0.438). The findings of this study are similar to the findings of a study in Kenya [19]. In contrast to the findings of our study, studies in Kenya and Swaziland found a statistically significant association between male partner involvement and infant HIV transmission rate (*P*<0.05), [23]–[24].

The major limitation of our study was a high loss to follow-up. More than half of the study participants were lost to follow-up by the 18 months postnatal period. This might have affected the results of the study by underestimation of HIV transmission rates among the children since the sample size became smaller in the course of the study due to loss to follow-up. Furthermore, the HIV status and health outcomes of the children who were lost to follow-up are unknown and might vary from those who completed follow-up and tested for HIV. The PMTCT registers had only data on children who died and on mother-child pairs who were lost to follow-up in the course of the study. No reasons were indicated as to why a large number of women were lost to follow-up. This highlights the need for a further study to establish the reasons of LTFU among PMTCT clients at Mwanza. It also calls for PMTCT providers to find ways of tracking clients who are lost to follow-up.

### Conclusions

This study has shown that male partner involvement increases the uptake of some PMTCT interventions by HIV positive women. The study was however, limited due to a small sample size as a result of high LTFU. Studies which are qualitative in nature are needed to explore the reasons for LTFU in PMTCT program. Longitudinal studies with big sample sizes are also warranted to evaluate the association between male partner involvement and uptake and outcomes of PMTCT interventions. Government, non-governmental organizations, health workers and all PMTCT stakeholders should explore multi-strategic, culturally tailored public health care models that would not only increase male partner involvement in PMTCT program, but also help to improve the effectiveness of PMTCT interventions.
